# Bis[bis­(2-ethyl-5-methyl-1*H*-imidazol-4-yl-κ*N* ^3^)methane](nitrato-κ^2^ *O*,*O*′)nickel(II) nitrate

**DOI:** 10.1107/S1600536811001000

**Published:** 2011-01-15

**Authors:** Ge Gao, Shu-Lin Mao, Xiao-Min Qian, Yang-Hui Luo, Jin-Feng Li

**Affiliations:** a Ordered Matter Science Research Center, College of Chemistry and Chemical Engineering, Southeast University, Nanjing 210096, People’s Republic of China

## Abstract

In the title compound, [Ni(NO_3_)(C_13_H_20_N_4_)_2_]NO_3_, the Ni^II^ ion shows a distorted octa­hedral geometry formed by four N atoms from two bis­(2-ethyl-5-methyl-1*H*-imidazol-4-yl)methane ligands and two O atoms from a chelating nitrate anion. Three ethyl groups in the complex cation and the O atoms of the uncoordinated nitrate anion are disordered over two sets of positions [occupancy ratios of 0.52 (3):0.48 (3) and 0.63 (3):0.37 (3), respectively]. In the crystal, inter­molecular N—H⋯O hydrogen bonds connect the complex cations into a zigzag chain along [010] and further N—H⋯O hydrogen bonds between the chains and the uncoordinated nitrate anions lead to layers parallel to (100).

## Related literature

For related structures, see: Davis *et al.* (2007 ▶); Liu & Zhang (2006 ▶); Martynowski *et al.* (2006 ▶); Policar *et al.* (1999 ▶).
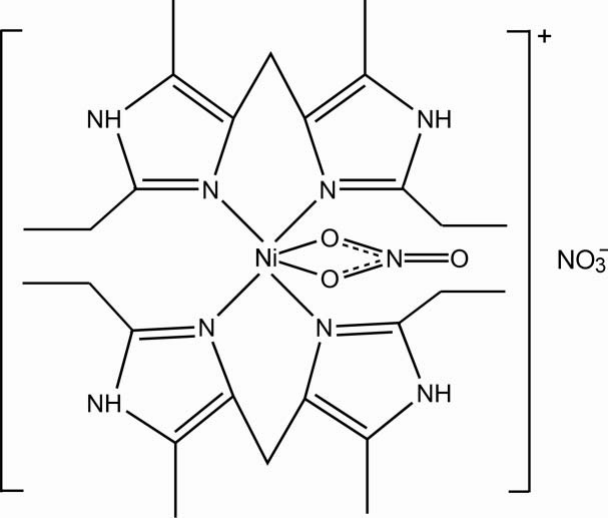


## Experimental

### 

#### Crystal data

[Ni(NO_3_)(C_13_H_20_N_4_)_2_]NO_3_
*M* *_r_* = 647.39
Monoclinic, 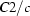

*a* = 37.163 (3) Å
*b* = 9.419 (1) Å
*c* = 19.0191 (18) Å
β = 104.470 (1)°
*V* = 6446.3 (11) Å^3^
*Z* = 8
Mo *K*α radiation
μ = 0.66 mm^−1^
*T* = 298 K
0.40 × 0.31 × 0.30 mm

#### Data collection

Rigaku SCXmini CCD diffractometer
Absorption correction: multi-scan (*CrystalClear*; Rigaku, 2005 ▶) *T*_min_ = 0.779, *T*_max_ = 0.828
15927 measured reflections
5688 independent reflections
2536 reflections with *I* > 2σ(*I*)
*R*_int_ = 0.077

#### Refinement

*R*[*F* ^2^ > 2σ(*F* ^2^)] = 0.053
*wR*(*F* ^2^) = 0.102
*S* = 1.08
5688 reflections
475 parameters
H-atom parameters constrained
Δρ_max_ = 0.29 e Å^−3^
Δρ_min_ = −0.22 e Å^−3^

### 

Data collection: *CrystalClear* (Rigaku, 2005 ▶); cell refinement: *CrystalClear*; data reduction: *CrystalClear*; program(s) used to solve structure: *SHELXS97* (Sheldrick, 2008 ▶); program(s) used to refine structure: *SHELXL97* (Sheldrick, 2008 ▶); molecular graphics: *SHELXTL* (Sheldrick, 2008 ▶); software used to prepare material for publication: *SHELXTL*.

## Supplementary Material

Crystal structure: contains datablocks I, global. DOI: 10.1107/S1600536811001000/hy2393sup1.cif

Structure factors: contains datablocks I. DOI: 10.1107/S1600536811001000/hy2393Isup2.hkl

Additional supplementary materials:  crystallographic information; 3D view; checkCIF report

## Figures and Tables

**Table 1:** Hydrogen-bond geometry (Å, °)

*D*—H⋯*A*	*D*—H	H⋯*A*	*D*⋯*A*	*D*—H⋯*A*
N2—H2⋯O4^i^	0.86	2.29	3.12 (2)	162
N2—H2⋯O5′^i^	0.86	1.88	2.67 (5)	152
N4—H4⋯O2^ii^	0.86	2.37	3.063 (5)	138
N6—H6⋯O6^iii^	0.86	2.05	2.83 (2)	152
N6—H6⋯O4′^iii^	0.86	2.18	2.98 (3)	155
N8—H8⋯O5^iv^	0.86	2.01	2.81 (3)	155

Symmetry codes: (i) 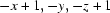; (ii) 
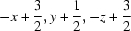
; (iii) 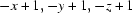; (iv) 
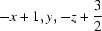
.

**Fig. 1.**
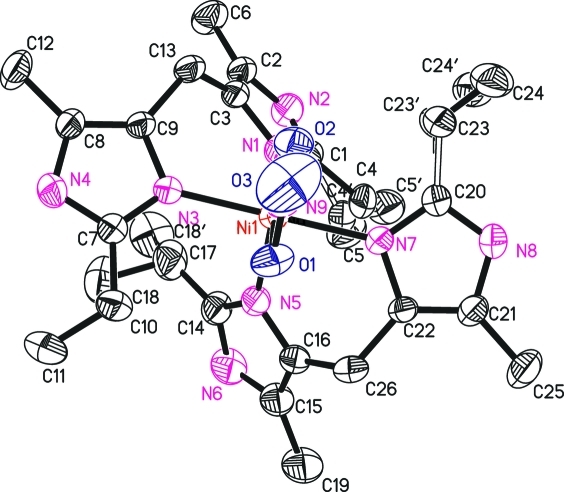
The molecular structure of the title compound. Displacement ellipsoids are drawn at the 30% probability level. The uncoordinated nitrate anion is not shown.

**Fig. 2.**
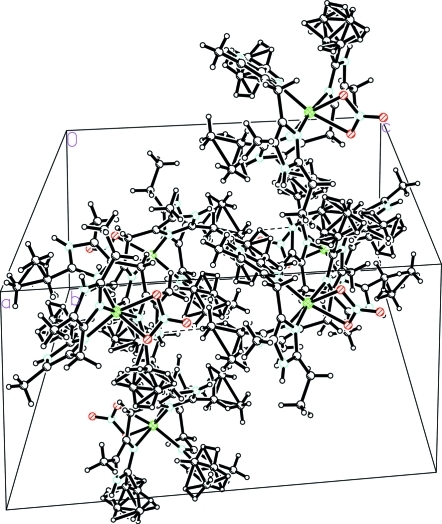
A packing diagram.

## supplementary crystallographic information

### Comment

Knowledge of the detailed coordination behavior of imidazoles and their limitation in the possible use in complexes with specific catalytic activity is of great current importance. Didentate nitrogen coordination to transition metal ions is a coordination mode, which is not uncommon in natural systems. Not many ligands with an analogous donor atom pattern are known. In fact, only a few crystal structures of the coordination compounds containing ligands with benzimidazole groups in the aforementioned arrangement have been reported (Davis *et al.*, 2007; Liu & Zhang, 2006; Martynowski *et al.*, 2006; Policar *et al.*, 1999). This paper describes the synthesis and structure of a coordination compound obtained from 5,5'-bis(2-ethyl-4-methylimidazol)methane and nickle(II) nitrate hexahydrate.

The asymmetric unit contains one Ni^II^ ion, two ligands, one coordinated nitrate anion and one uncoordinated nitrate anion. The Ni^II^ ion shows a distorted octahedral geometry formed by four N atoms from two ligands and two O atoms from one nitrate (Fig. 1). The dihedral angles formed by two imidazole rings of the same ligand are 50.6 (2) and 55.0 (2)°. In the crystal structure, intermolecular N—H···O hydrogen bonds (Table 1) connect the complex cations into a zigzag chain along [0 1 0] and N—H···O hydrogen bonds between the chains and the uncoordinated nitrate anions lead to a layer parallel to (1 0 0).

### Experimental

All chemicals used (reagent grade) were commercially available. To a methanol solution (3 ml) of nickel(II) nitrate hexahydrate (0.113 g, 0.2 mmol), a methanol solution (3 ml) of 5,5'-bis(2-ethyl-4-methylimidazol)methane (0.058 g, 0.2 mmol) was added dropwise with stirring for about 30 min, and then filtered. The filtrate was slowly evaporated at room temperature over several days and blue crystals suitable for X-ray analysis were obtained.

### Refinement

H atoms were positioned geometrically and refined as riding atoms, with C—H = 0.97 (CH_2_) and 0.96 (CH_3_) Å and N—H = 0.86 Å and with *U*_iso_(H) = 1.2(1.5 for methyl)*U*_eq_(C,N).

### Crystal data

**Table:**

[Ni(NO_3_)(C_13_H_20_N_4_)_2_]NO_3_	*F*(000) = 2736
*M**_r_* = 647.39	*D*_x_ = 1.334 Mg m^−^^3^
Monoclinic, *C*2/*c*	Mo *K*α radiation, λ = 0.71073 Å
Hall symbol: -C 2yc	Cell parameters from 1945 reflections
*a* = 37.163 (3) Å	θ = 2.2–18.0°
*b* = 9.419 (1) Å	µ = 0.66 mm^−^^1^
*c* = 19.0191 (18) Å	*T* = 298 K
β = 104.470 (1)°	Prism, blue
*V* = 6446.3 (11) Å^3^	0.40 × 0.31 × 0.30 mm
*Z* = 8	

### Data collection

**Table:**

Rigaku SCXmini CCD diffractometer	5688 independent reflections
Radiation source: fine-focus sealed tube	2536 reflections with *I* > 2σ(*I*)
graphite	*R*_int_ = 0.077
Detector resolution: 8.192 pixels mm^-1^	θ_max_ = 25.0°, θ_min_ = 2.2°
ω scans	*h* = −44→35
Absorption correction: multi-scan (*CrystalClear*; Rigaku, 2005)	*k* = −11→11
*T*_min_ = 0.779, *T*_max_ = 0.828	*l* = −22→20
15927 measured reflections	

### Refinement

**Table:**

Refinement on *F*^2^	Primary atom site location: structure-invariant direct methods
Least-squares matrix: full	Secondary atom site location: difference Fourier map
*R*[*F*^2^ > 2σ(*F*^2^)] = 0.053	Hydrogen site location: inferred from neighbouring sites
*wR*(*F*^2^) = 0.102	H-atom parameters constrained
*S* = 1.08	*w* = 1/[σ^2^(*F*_o_^2^) + (0.0152*P*)^2^] where *P* = (*F*_o_^2^ + 2*F*_c_^2^)/3
5688 reflections	(Δ/σ)_max_ = 0.001
475 parameters	Δρ_max_ = 0.29 e Å^−^^3^
0 restraints	Δρ_min_ = −0.22 e Å^−^^3^

### Fractional atomic coordinates and isotropic or equivalent isotropic displacement parameters (Å^2^)

**Table:**

	*x*	*y*	*z*	*U*_iso_*/*U*_eq_	Occ. (<1)
Ni1	0.650545 (16)	0.51105 (6)	0.71506 (3)	0.0569 (2)	
N1	0.63927 (12)	0.3681 (4)	0.6308 (2)	0.0650 (11)	
N2	0.61323 (12)	0.2495 (4)	0.5326 (2)	0.0771 (13)	
H2	0.5964	0.2103	0.4990	0.092*	
N3	0.69444 (9)	0.5929 (4)	0.67886 (17)	0.0563 (10)	
N4	0.73519 (11)	0.7267 (4)	0.64743 (19)	0.0677 (11)	
H4	0.7479	0.8006	0.6427	0.081*	
N5	0.61253 (10)	0.6598 (4)	0.6652 (2)	0.0630 (11)	
N6	0.57373 (13)	0.7919 (5)	0.5875 (2)	0.0904 (14)	
H6	0.5633	0.8351	0.5479	0.109*	
N7	0.61273 (10)	0.4337 (4)	0.76797 (17)	0.0634 (11)	
N8	0.57560 (12)	0.3043 (5)	0.8130 (2)	0.0856 (13)	
H8	0.5658	0.2309	0.8276	0.103*	
N9	0.69820 (13)	0.5096 (6)	0.8410 (3)	0.0883 (14)	
N10	0.44513 (16)	0.0136 (12)	0.5796 (5)	0.092 (2)	
O1	0.67689 (9)	0.6134 (4)	0.82047 (16)	0.0786 (11)	
O2	0.69436 (10)	0.4045 (4)	0.79776 (19)	0.0850 (11)	
O3	0.72186 (12)	0.5057 (6)	0.8983 (2)	0.157 (2)	
O4	0.4328 (3)	−0.091 (2)	0.6047 (10)	0.133 (7)	0.63 (3)
O5	0.4581 (7)	0.121 (3)	0.6057 (16)	0.144 (11)	0.63 (3)
O6	0.4473 (7)	−0.013 (2)	0.5157 (13)	0.110 (5)	0.63 (3)
O4'	0.4538 (12)	0.138 (4)	0.5696 (14)	0.103 (10)	0.37 (3)
O5'	0.4400 (13)	−0.071 (5)	0.535 (3)	0.17 (2)	0.37 (3)
O6'	0.4477 (9)	0.001 (3)	0.6470 (15)	0.104 (12)	0.37 (3)
C1	0.60730 (16)	0.3215 (5)	0.5906 (3)	0.0677 (14)	
C2	0.65083 (16)	0.2499 (5)	0.5368 (3)	0.0678 (15)	
C3	0.66668 (14)	0.3228 (5)	0.5976 (3)	0.0605 (13)	
C4	0.5691 (12)	0.329 (3)	0.604 (2)	0.078 (7)	0.72 (5)
H4A	0.5695	0.3937	0.6439	0.094*	0.72 (5)
H4B	0.5622	0.2356	0.6183	0.094*	0.72 (5)
C5	0.5402 (5)	0.379 (3)	0.5363 (10)	0.112 (7)	0.72 (5)
H5A	0.5453	0.4750	0.5257	0.167*	0.72 (5)
H5B	0.5158	0.3730	0.5449	0.167*	0.72 (5)
H5C	0.5412	0.3190	0.4960	0.167*	0.72 (5)
C4'	0.569 (3)	0.380 (9)	0.592 (6)	0.074 (16)	0.28 (5)
H4'1	0.5662	0.4709	0.5673	0.089*	0.28 (5)
H4'2	0.5691	0.3966	0.6420	0.089*	0.28 (5)
C5'	0.5359 (12)	0.286 (8)	0.557 (3)	0.105 (17)	0.28 (5)
H5'1	0.5138	0.3433	0.5456	0.158*	0.28 (5)
H5'2	0.5334	0.2125	0.5902	0.158*	0.28 (5)
H5'3	0.5396	0.2448	0.5132	0.158*	0.28 (5)
C6	0.66657 (14)	0.1795 (5)	0.4803 (2)	0.0977 (18)	
H6A	0.6832	0.2438	0.4650	0.147*	
H6B	0.6467	0.1540	0.4394	0.147*	
H6C	0.6799	0.0956	0.5005	0.147*	
C7	0.70839 (13)	0.7219 (5)	0.6826 (2)	0.0598 (13)	
C8	0.73922 (13)	0.5930 (5)	0.6197 (2)	0.0623 (13)	
C9	0.71433 (12)	0.5125 (5)	0.6403 (2)	0.0542 (11)	
C10	0.69670 (13)	0.8465 (5)	0.7201 (2)	0.0781 (15)	
H10A	0.6701	0.8399	0.7152	0.094*	
H10B	0.7088	0.8398	0.7715	0.094*	
C11	0.70486 (15)	0.9881 (5)	0.6940 (3)	0.1082 (19)	
H11A	0.7312	0.9984	0.7007	0.162*	
H11B	0.6959	1.0605	0.7209	0.162*	
H11C	0.6927	0.9973	0.6433	0.162*	
C12	0.76670 (13)	0.5625 (5)	0.5767 (2)	0.0908 (17)	
H12A	0.7908	0.5492	0.6089	0.136*	
H12B	0.7675	0.6407	0.5447	0.136*	
H12C	0.7595	0.4778	0.5486	0.136*	
C13	0.70651 (12)	0.3557 (4)	0.6293 (2)	0.0649 (13)	
H13A	0.7210	0.3184	0.5975	0.078*	
H13B	0.7146	0.3078	0.6757	0.078*	
C14	0.60378 (16)	0.7086 (6)	0.5977 (3)	0.0754 (16)	
C15	0.56248 (15)	0.7971 (6)	0.6504 (3)	0.0794 (16)	
C16	0.58627 (14)	0.7145 (5)	0.6974 (3)	0.0638 (14)	
C17	0.62176 (18)	0.6744 (7)	0.5375 (3)	0.104 (2)	
H17A	0.6398	0.5993	0.5538	0.125*	0.802 (10)
H17B	0.6029	0.6381	0.4966	0.125*	0.802 (10)
H17C	0.6399	0.6040	0.5607	0.125*	0.198 (10)
H17D	0.6362	0.7600	0.5375	0.125*	0.198 (10)
C18	0.6403 (2)	0.7937 (9)	0.5130 (4)	0.124 (4)	0.802 (10)
H18A	0.6222	0.8641	0.4914	0.185*	0.802 (10)
H18B	0.6530	0.7613	0.4777	0.185*	0.802 (10)
H18C	0.6580	0.8344	0.5536	0.185*	0.802 (10)
C18'	0.6126 (8)	0.638 (4)	0.4591 (18)	0.129 (16)	0.198 (10)
H18D	0.6127	0.5370	0.4535	0.194*	0.198 (10)
H18E	0.6307	0.6798	0.4372	0.194*	0.198 (10)
H18F	0.5884	0.6746	0.4359	0.194*	0.198 (10)
C19	0.52842 (14)	0.8794 (5)	0.6555 (3)	0.111 (2)	
H19A	0.5067	0.8344	0.6260	0.167*	
H19B	0.5301	0.9747	0.6387	0.167*	
H19C	0.5268	0.8814	0.7051	0.167*	
C20	0.60526 (16)	0.3029 (6)	0.7853 (3)	0.0764 (16)	
C21	0.56334 (14)	0.4411 (6)	0.8146 (2)	0.0728 (15)	
C22	0.58615 (12)	0.5196 (5)	0.7864 (2)	0.0566 (12)	
C23	0.6206 (8)	0.169 (3)	0.7596 (15)	0.077 (7)	0.52 (3)
H23A	0.6020	0.1286	0.7195	0.092*	0.52 (3)
H23B	0.6422	0.1931	0.7420	0.092*	0.52 (3)
C24	0.6315 (6)	0.0605 (18)	0.8191 (14)	0.104 (7)	0.52 (3)
H24A	0.6461	0.1052	0.8623	0.155*	0.52 (3)
H24B	0.6458	−0.0132	0.8042	0.155*	0.52 (3)
H24C	0.6095	0.0202	0.8289	0.155*	0.52 (3)
C23'	0.6332 (9)	0.181 (4)	0.7946 (13)	0.073 (8)	0.48 (3)
H23C	0.6539	0.2053	0.7743	0.087*	0.48 (3)
H23D	0.6426	0.1566	0.8454	0.087*	0.48 (3)
C24'	0.6105 (6)	0.060 (2)	0.7527 (18)	0.109 (9)	0.48 (3)
H24D	0.5887	0.0448	0.7702	0.164*	0.48 (3)
H24E	0.6252	−0.0253	0.7594	0.164*	0.48 (3)
H24F	0.6032	0.0831	0.7020	0.164*	0.48 (3)
C25	0.53078 (13)	0.4778 (5)	0.8438 (2)	0.1056 (19)	
H25A	0.5295	0.5790	0.8489	0.158*	
H25B	0.5335	0.4336	0.8903	0.158*	
H25C	0.5084	0.4445	0.8110	0.158*	
C26	0.58718 (13)	0.6773 (5)	0.7746 (2)	0.0682 (14)	
H26A	0.5661	0.7209	0.7875	0.082*	
H26B	0.6096	0.7159	0.8063	0.082*	

### Atomic displacement parameters (Å^2^)

**Table:**

	*U*^11^	*U*^22^	*U*^33^	*U*^12^	*U*^13^	*U*^23^
Ni1	0.0592 (4)	0.0539 (4)	0.0624 (4)	0.0103 (4)	0.0243 (3)	0.0046 (3)
N1	0.060 (3)	0.065 (3)	0.076 (3)	−0.005 (2)	0.028 (3)	−0.005 (2)
N2	0.074 (3)	0.081 (3)	0.078 (3)	−0.010 (3)	0.021 (3)	−0.016 (2)
N3	0.059 (3)	0.047 (2)	0.068 (3)	0.001 (2)	0.025 (2)	−0.002 (2)
N4	0.069 (3)	0.057 (3)	0.080 (3)	−0.015 (2)	0.023 (2)	−0.006 (2)
N5	0.063 (3)	0.063 (3)	0.066 (3)	0.010 (2)	0.022 (3)	0.013 (2)
N6	0.083 (4)	0.092 (4)	0.089 (4)	0.023 (3)	0.007 (3)	0.038 (3)
N7	0.074 (3)	0.056 (3)	0.072 (3)	0.012 (2)	0.040 (2)	0.010 (2)
N8	0.096 (4)	0.074 (3)	0.104 (3)	0.009 (3)	0.057 (3)	0.026 (3)
N9	0.076 (4)	0.122 (5)	0.067 (4)	0.017 (4)	0.019 (3)	0.015 (4)
N10	0.075 (4)	0.101 (8)	0.095 (7)	0.013 (5)	0.013 (5)	−0.024 (8)
O1	0.075 (3)	0.090 (3)	0.070 (2)	0.027 (2)	0.016 (2)	−0.002 (2)
O2	0.078 (3)	0.081 (3)	0.099 (3)	0.031 (2)	0.027 (2)	0.026 (2)
O3	0.121 (4)	0.255 (6)	0.078 (3)	0.061 (4)	−0.008 (3)	0.028 (4)
O4	0.121 (6)	0.179 (14)	0.097 (11)	−0.062 (7)	0.023 (6)	0.051 (11)
O5	0.130 (11)	0.14 (2)	0.17 (2)	0.016 (11)	0.048 (16)	−0.08 (2)
O6	0.146 (11)	0.075 (10)	0.097 (8)	−0.001 (8)	0.007 (6)	0.019 (8)
O4'	0.134 (14)	0.088 (19)	0.078 (15)	0.012 (12)	0.007 (14)	0.022 (15)
O5'	0.15 (2)	0.12 (3)	0.19 (5)	0.00 (2)	−0.02 (3)	−0.06 (3)
O6'	0.14 (2)	0.097 (16)	0.102 (16)	0.037 (13)	0.077 (15)	0.035 (13)
C1	0.065 (4)	0.071 (4)	0.074 (4)	−0.003 (3)	0.030 (4)	−0.005 (3)
C2	0.069 (4)	0.060 (4)	0.082 (4)	−0.003 (3)	0.033 (4)	−0.012 (3)
C3	0.060 (4)	0.048 (3)	0.081 (4)	−0.005 (3)	0.030 (3)	−0.009 (3)
C4	0.065 (10)	0.082 (17)	0.093 (17)	−0.011 (15)	0.030 (11)	0.006 (13)
C5	0.075 (10)	0.135 (17)	0.124 (11)	0.025 (10)	0.023 (8)	0.008 (10)
C4'	0.07 (3)	0.07 (4)	0.08 (4)	0.00 (4)	0.03 (2)	0.01 (3)
C5'	0.10 (3)	0.10 (3)	0.12 (3)	−0.01 (2)	0.02 (2)	−0.02 (2)
C6	0.103 (5)	0.096 (4)	0.107 (4)	−0.007 (4)	0.051 (4)	−0.038 (3)
C7	0.058 (3)	0.055 (3)	0.070 (3)	0.000 (3)	0.022 (3)	−0.001 (3)
C8	0.060 (3)	0.061 (3)	0.073 (3)	−0.004 (3)	0.031 (3)	−0.007 (3)
C9	0.055 (3)	0.048 (3)	0.064 (3)	0.001 (3)	0.024 (3)	−0.010 (3)
C10	0.082 (4)	0.055 (3)	0.102 (4)	−0.001 (3)	0.032 (3)	−0.014 (3)
C11	0.139 (5)	0.055 (4)	0.142 (5)	0.013 (4)	0.056 (4)	−0.002 (4)
C12	0.076 (4)	0.107 (5)	0.106 (4)	−0.016 (3)	0.055 (3)	−0.020 (3)
C13	0.063 (4)	0.056 (3)	0.084 (4)	0.001 (3)	0.034 (3)	−0.016 (3)
C14	0.072 (4)	0.084 (4)	0.074 (4)	0.014 (3)	0.024 (4)	0.025 (3)
C15	0.063 (4)	0.076 (4)	0.096 (5)	0.014 (3)	0.015 (4)	0.019 (4)
C16	0.061 (4)	0.056 (3)	0.076 (4)	0.012 (3)	0.020 (3)	0.012 (3)
C17	0.103 (5)	0.115 (6)	0.098 (6)	0.011 (4)	0.030 (5)	0.039 (4)
C18	0.097 (6)	0.155 (9)	0.122 (7)	−0.006 (6)	0.034 (5)	0.041 (6)
C18'	0.11 (3)	0.15 (4)	0.10 (3)	0.01 (2)	−0.03 (2)	0.03 (2)
C19	0.084 (4)	0.097 (5)	0.149 (5)	0.040 (4)	0.023 (4)	0.034 (4)
C20	0.087 (5)	0.063 (4)	0.092 (4)	0.012 (4)	0.047 (4)	0.018 (3)
C21	0.077 (4)	0.077 (4)	0.076 (4)	0.018 (3)	0.041 (3)	0.013 (3)
C22	0.065 (3)	0.053 (3)	0.058 (3)	0.016 (3)	0.027 (3)	0.007 (3)
C23	0.085 (19)	0.059 (13)	0.090 (19)	0.010 (12)	0.029 (14)	0.016 (17)
C24	0.123 (13)	0.087 (13)	0.103 (17)	0.040 (9)	0.032 (13)	0.023 (9)
C23'	0.08 (2)	0.062 (13)	0.079 (16)	0.015 (14)	0.031 (15)	0.014 (16)
C24'	0.101 (14)	0.081 (13)	0.15 (2)	0.003 (10)	0.046 (16)	−0.011 (12)
C25	0.098 (4)	0.127 (5)	0.112 (4)	0.027 (4)	0.066 (4)	0.022 (4)
C26	0.066 (4)	0.061 (4)	0.082 (4)	0.018 (3)	0.025 (3)	−0.001 (3)

### Geometric parameters (Å, °)

**Table:**

Ni1—N5	2.046 (4)	C8—C9	1.328 (5)
Ni1—N1	2.055 (4)	C8—C12	1.488 (5)
Ni1—N7	2.055 (3)	C9—C13	1.510 (5)
Ni1—N3	2.071 (3)	C10—C11	1.482 (5)
Ni1—O2	2.203 (3)	C10—H10A	0.9700
Ni1—O1	2.219 (3)	C10—H10B	0.9700
N1—C1	1.317 (5)	C11—H11A	0.9600
N1—C3	1.393 (5)	C11—H11B	0.9600
N2—C1	1.359 (5)	C11—H11C	0.9600
N2—C2	1.380 (5)	C12—H12A	0.9600
N2—H2	0.8600	C12—H12B	0.9600
N3—C7	1.316 (5)	C12—H12C	0.9600
N3—C9	1.389 (4)	C13—H13A	0.9700
N4—C7	1.332 (5)	C13—H13B	0.9700
N4—C8	1.387 (5)	C14—C17	1.497 (6)
N4—H4	0.8600	C15—C16	1.339 (6)
N5—C14	1.326 (5)	C15—C19	1.508 (6)
N5—C16	1.376 (5)	C16—C26	1.500 (5)
N6—C14	1.339 (5)	C17—C18	1.455 (8)
N6—C15	1.362 (5)	C17—C18'	1.48 (3)
N6—H6	0.8600	C17—H17A	0.9700
N7—C20	1.323 (5)	C17—H17B	0.9700
N7—C22	1.388 (5)	C17—H17C	0.9700
N8—C20	1.335 (5)	C17—H17D	0.9700
N8—C21	1.370 (5)	C18—H17D	0.6134
N8—H8	0.8600	C18—H18A	0.9600
N9—O3	1.218 (5)	C18—H18B	0.9600
N9—O1	1.257 (5)	C18—H18C	0.9600
N9—O2	1.272 (5)	C18'—H18D	0.9600
N10—O5'	1.14 (5)	C18'—H18E	0.9600
N10—O5	1.17 (3)	C18'—H18F	0.9600
N10—O4	1.230 (16)	C19—H19A	0.9600
N10—O4'	1.25 (5)	C19—H19B	0.9600
N10—O6	1.26 (3)	C19—H19C	0.9600
N10—O6'	1.27 (2)	C20—C23	1.51 (3)
C1—C4	1.51 (4)	C20—C23'	1.53 (3)
C1—C4'	1.52 (11)	C21—C22	1.333 (6)
C2—C3	1.348 (6)	C21—C25	1.493 (5)
C2—C6	1.499 (5)	C22—C26	1.504 (5)
C3—C13	1.485 (5)	C23—C24	1.50 (5)
C4—C5	1.53 (6)	C23—H23A	0.9700
C4—H4A	0.9700	C23—H23B	0.9700
C4—H4B	0.9700	C24—H24A	0.9600
C5—H5A	0.9600	C24—H24B	0.9600
C5—H5B	0.9600	C24—H24C	0.9600
C5—H5C	0.9600	C23'—C24'	1.52 (5)
C4'—C5'	1.53 (15)	C23'—H23C	0.9700
C4'—H4'1	0.9700	C23'—H23D	0.9700
C4'—H4'2	0.9700	C24'—H24D	0.9600
C5'—H5'1	0.9600	C24'—H24E	0.9600
C5'—H5'2	0.9600	C24'—H24F	0.9600
C5'—H5'3	0.9600	C25—H25A	0.9600
C6—H6A	0.9600	C25—H25B	0.9600
C6—H6B	0.9600	C25—H25C	0.9600
C6—H6C	0.9600	C26—H26A	0.9700
C7—C10	1.493 (5)	C26—H26B	0.9700
			
N5—Ni1—N1	95.98 (16)	H10A—C10—H10B	107.4
N5—Ni1—N7	89.56 (15)	C10—C11—H11A	109.5
N1—Ni1—N7	96.82 (15)	C10—C11—H11B	109.5
N5—Ni1—N3	95.79 (14)	H11A—C11—H11B	109.5
N1—Ni1—N3	90.50 (15)	C10—C11—H11C	109.5
N7—Ni1—N3	170.46 (14)	H11A—C11—H11C	109.5
N5—Ni1—O2	160.78 (15)	H11B—C11—H11C	109.5
N1—Ni1—O2	103.24 (15)	C8—C12—H12A	109.5
N7—Ni1—O2	87.87 (13)	C8—C12—H12B	109.5
N3—Ni1—O2	84.48 (13)	H12A—C12—H12B	109.5
N5—Ni1—O1	102.65 (14)	C8—C12—H12C	109.5
N1—Ni1—O1	161.30 (15)	H12A—C12—H12C	109.5
N7—Ni1—O1	85.00 (13)	H12B—C12—H12C	109.5
N3—Ni1—O1	86.12 (13)	C3—C13—C9	113.4 (4)
O2—Ni1—O1	58.15 (13)	C3—C13—H13A	108.9
C1—N1—C3	106.9 (4)	C9—C13—H13A	108.9
C1—N1—Ni1	130.5 (4)	C3—C13—H13B	108.9
C3—N1—Ni1	121.6 (4)	C9—C13—H13B	108.9
C1—N2—C2	108.7 (5)	H13A—C13—H13B	107.7
C1—N2—H2	125.7	N5—C14—N6	109.7 (5)
C2—N2—H2	125.7	N5—C14—C17	128.1 (5)
C7—N3—C9	106.1 (4)	N6—C14—C17	122.1 (5)
C7—N3—Ni1	131.1 (3)	C16—C15—N6	105.6 (5)
C9—N3—Ni1	122.7 (3)	C16—C15—C19	132.9 (6)
C7—N4—C8	109.0 (4)	N6—C15—C19	121.5 (5)
C7—N4—H4	125.5	C15—C16—N5	110.1 (4)
C8—N4—H4	125.5	C15—C16—C26	129.7 (5)
C14—N5—C16	105.8 (4)	N5—C16—C26	120.2 (4)
C14—N5—Ni1	131.3 (4)	C18—C17—C18'	82.1 (12)
C16—N5—Ni1	122.3 (3)	C18—C17—C14	114.6 (6)
C14—N6—C15	108.8 (5)	C18'—C17—C14	141.5 (13)
C14—N6—H6	125.6	C18—C17—H17A	108.6
C15—N6—H6	125.6	C18'—C17—H17A	96.9
C20—N7—C22	105.8 (4)	C14—C17—H17A	108.6
C20—N7—Ni1	131.6 (4)	C18—C17—H17B	108.6
C22—N7—Ni1	122.3 (3)	C14—C17—H17B	108.6
C20—N8—C21	109.2 (4)	H17A—C17—H17B	107.6
C20—N8—H8	125.4	C18—C17—H17C	110.0
C21—N8—H8	125.4	C18'—C17—H17C	105.0
O3—N9—O1	124.0 (6)	C14—C17—H17C	101.1
O3—N9—O2	119.7 (6)	H17B—C17—H17C	113.9
O1—N9—O2	116.4 (5)	C18'—C17—H17D	100.3
O5'—N10—O5	152 (3)	C14—C17—H17D	100.1
O5'—N10—O4	74 (3)	H17A—C17—H17D	105.5
O5—N10—O4	132.6 (17)	H17B—C17—H17D	125.6
O5'—N10—O4'	123 (3)	H17C—C17—H17D	104.2
O4—N10—O4'	161.5 (19)	C17—C18—H18A	109.5
O5—N10—O6	117.3 (19)	H17D—C18—H18A	113.7
O4—N10—O6	109.5 (16)	C17—C18—H18B	109.5
O4'—N10—O6	87.6 (19)	H17D—C18—H18B	128.6
O5'—N10—O6'	130 (3)	C17—C18—H18C	109.5
O5—N10—O6'	74.5 (16)	H17D—C18—H18C	81.0
O4—N10—O6'	58.4 (13)	C17—C18'—H18D	109.5
O4'—N10—O6'	107 (2)	C17—C18'—H18E	109.5
O6—N10—O6'	161.7 (15)	H18D—C18'—H18E	109.5
N9—O1—Ni1	92.5 (3)	C17—C18'—H18F	109.5
N9—O2—Ni1	92.9 (3)	H18D—C18'—H18F	109.5
N1—C1—N2	109.4 (5)	H18E—C18'—H18F	109.5
N1—C1—C4	129.6 (18)	C15—C19—H19A	109.5
N2—C1—C4	120.8 (18)	C15—C19—H19B	109.5
N1—C1—C4'	125 (5)	H19A—C19—H19B	109.5
N2—C1—C4'	122 (5)	C15—C19—H19C	109.5
C3—C2—N2	105.6 (4)	H19A—C19—H19C	109.5
C3—C2—C6	132.4 (5)	H19B—C19—H19C	109.5
N2—C2—C6	121.9 (5)	N7—C20—N8	109.6 (5)
C2—C3—N1	109.4 (4)	N7—C20—C23	125.1 (13)
C2—C3—C13	129.2 (5)	N8—C20—C23	123.4 (13)
N1—C3—C13	121.4 (5)	N7—C20—C23'	123.4 (15)
C1—C4—C5	111 (3)	N8—C20—C23'	124.4 (14)
C1—C4—H4A	109.4	C22—C21—N8	105.4 (4)
C5—C4—H4A	109.4	C22—C21—C25	132.6 (5)
C1—C4—H4B	109.4	N8—C21—C25	122.1 (5)
C5—C4—H4B	109.4	C21—C22—N7	110.0 (4)
H4A—C4—H4B	108.0	C21—C22—C26	130.6 (4)
C1—C4'—C5'	116 (7)	N7—C22—C26	119.4 (4)
C1—C4'—H4'1	107.0	C24—C23—C20	112 (2)
C5'—C4'—H4'1	109.0	C24—C23—H23A	109.2
C1—C4'—H4'2	108.2	C20—C23—H23A	109.2
C5'—C4'—H4'2	108.2	C24—C23—H23B	109.2
H4'1—C4'—H4'2	107.4	C20—C23—H23B	109.2
C4'—C5'—H5'1	109.5	H23A—C23—H23B	107.9
C4'—C5'—H5'2	109.5	C24'—C23'—C20	103 (2)
H5'1—C5'—H5'2	109.5	C24'—C23'—H23C	111.1
C4'—C5'—H5'3	109.5	C20—C23'—H23C	111.1
H5'1—C5'—H5'3	109.5	C24'—C23'—H23D	111.1
H5'2—C5'—H5'3	109.5	C20—C23'—H23D	111.1
C2—C6—H6A	109.5	H23C—C23'—H23D	109.0
C2—C6—H6B	109.5	C23'—C24'—H24D	109.5
H6A—C6—H6B	109.5	C23'—C24'—H24E	109.5
C2—C6—H6C	109.5	H24D—C24'—H24E	109.5
H6A—C6—H6C	109.5	C23'—C24'—H24F	109.5
H6B—C6—H6C	109.5	H24D—C24'—H24F	109.5
N3—C7—N4	109.7 (4)	H24E—C24'—H24F	109.5
N3—C7—C10	126.4 (5)	C21—C25—H25A	109.5
N4—C7—C10	123.8 (5)	C21—C25—H25B	109.5
C9—C8—N4	104.9 (4)	H25A—C25—H25B	109.5
C9—C8—C12	132.5 (5)	C21—C25—H25C	109.5
N4—C8—C12	122.6 (5)	H25A—C25—H25C	109.5
C8—C9—N3	110.2 (4)	H25B—C25—H25C	109.5
C8—C9—C13	129.9 (4)	C16—C26—C22	112.5 (4)
N3—C9—C13	119.8 (4)	C16—C26—H26A	109.1
C11—C10—C7	116.0 (4)	C22—C26—H26A	109.1
C11—C10—H10A	108.3	C16—C26—H26B	109.1
C7—C10—H10A	108.3	C22—C26—H26B	109.1
C11—C10—H10B	108.3	H26A—C26—H26B	107.8
C7—C10—H10B	108.3		
			
N5—Ni1—N1—C1	−45.8 (5)	N2—C1—C4'—C5'	38 (7)
N7—Ni1—N1—C1	44.4 (5)	C4—C1—C4'—C5'	−54 (15)
N3—Ni1—N1—C1	−141.7 (4)	C9—N3—C7—N4	1.2 (5)
O2—Ni1—N1—C1	133.8 (4)	Ni1—N3—C7—N4	−175.9 (3)
O1—Ni1—N1—C1	139.0 (5)	C9—N3—C7—C10	−178.7 (4)
N5—Ni1—N1—C3	121.6 (3)	Ni1—N3—C7—C10	4.2 (7)
N7—Ni1—N1—C3	−148.2 (3)	C8—N4—C7—N3	−0.6 (5)
N3—Ni1—N1—C3	25.7 (3)	C8—N4—C7—C10	179.3 (4)
O2—Ni1—N1—C3	−58.7 (3)	C7—N4—C8—C9	−0.4 (5)
O1—Ni1—N1—C3	−53.6 (6)	C7—N4—C8—C12	179.3 (4)
N5—Ni1—N3—C7	52.5 (4)	N4—C8—C9—N3	1.1 (5)
N1—Ni1—N3—C7	148.5 (4)	C12—C8—C9—N3	−178.5 (4)
O2—Ni1—N3—C7	−108.2 (4)	N4—C8—C9—C13	−177.1 (4)
O1—Ni1—N3—C7	−49.9 (4)	C12—C8—C9—C13	3.2 (8)
N5—Ni1—N3—C9	−124.2 (3)	C7—N3—C9—C8	−1.5 (5)
N1—Ni1—N3—C9	−28.1 (3)	Ni1—N3—C9—C8	175.9 (3)
O2—Ni1—N3—C9	75.1 (3)	C7—N3—C9—C13	177.0 (4)
O1—Ni1—N3—C9	133.5 (3)	Ni1—N3—C9—C13	−5.6 (5)
N1—Ni1—N5—C14	−42.6 (5)	N3—C7—C10—C11	−156.6 (5)
N7—Ni1—N5—C14	−139.4 (4)	N4—C7—C10—C11	23.5 (7)
N3—Ni1—N5—C14	48.6 (5)	C2—C3—C13—C9	123.6 (5)
O2—Ni1—N5—C14	138.4 (5)	N1—C3—C13—C9	−56.5 (6)
O1—Ni1—N5—C14	135.9 (4)	C8—C9—C13—C3	−128.8 (5)
N1—Ni1—N5—C16	127.2 (4)	N3—C9—C13—C3	53.1 (5)
N7—Ni1—N5—C16	30.4 (4)	C16—N5—C14—N6	0.8 (6)
N3—Ni1—N5—C16	−141.7 (3)	Ni1—N5—C14—N6	171.8 (3)
O2—Ni1—N5—C16	−51.8 (6)	C16—N5—C14—C17	−175.7 (6)
O1—Ni1—N5—C16	−54.4 (4)	Ni1—N5—C14—C17	−4.7 (9)
N5—Ni1—N7—C20	142.7 (5)	C15—N6—C14—N5	−0.2 (6)
N1—Ni1—N7—C20	46.7 (5)	C15—N6—C14—C17	176.6 (5)
O2—Ni1—N7—C20	−56.4 (5)	C14—N6—C15—C16	−0.5 (6)
O1—Ni1—N7—C20	−114.6 (5)	C14—N6—C15—C19	−178.1 (5)
N5—Ni1—N7—C22	−30.3 (3)	N6—C15—C16—N5	1.0 (6)
N1—Ni1—N7—C22	−126.2 (3)	C19—C15—C16—N5	178.2 (5)
O2—Ni1—N7—C22	130.7 (3)	N6—C15—C16—C26	−178.5 (5)
O1—Ni1—N7—C22	72.5 (3)	C19—C15—C16—C26	−1.3 (10)
O3—N9—O1—Ni1	177.4 (5)	C14—N5—C16—C15	−1.1 (6)
O2—N9—O1—Ni1	−2.0 (5)	Ni1—N5—C16—C15	−173.2 (3)
N5—Ni1—O1—N9	−179.8 (3)	C14—N5—C16—C26	178.4 (4)
N1—Ni1—O1—N9	−4.7 (6)	Ni1—N5—C16—C26	6.4 (6)
N7—Ni1—O1—N9	91.8 (3)	N5—C14—C17—C18	−114.8 (6)
N3—Ni1—O1—N9	−84.7 (3)	N6—C14—C17—C18	69.0 (8)
O2—Ni1—O1—N9	1.2 (3)	N5—C14—C17—C18'	136 (2)
O3—N9—O2—Ni1	−177.4 (4)	N6—C14—C17—C18'	−41 (2)
O1—N9—O2—Ni1	2.0 (5)	C22—N7—C20—N8	0.2 (5)
N5—Ni1—O2—N9	−4.1 (6)	Ni1—N7—C20—N8	−173.6 (3)
N1—Ni1—O2—N9	176.9 (3)	C22—N7—C20—C23	164.8 (13)
N7—Ni1—O2—N9	−86.6 (3)	Ni1—N7—C20—C23	−9.0 (15)
N3—Ni1—O2—N9	87.7 (3)	C22—N7—C20—C23'	−162.1 (12)
O1—Ni1—O2—N9	−1.2 (3)	Ni1—N7—C20—C23'	24.1 (14)
C3—N1—C1—N2	−0.6 (5)	C21—N8—C20—N7	−0.6 (6)
Ni1—N1—C1—N2	168.2 (3)	C21—N8—C20—C23	−165.5 (13)
C3—N1—C1—C4	174.2 (16)	C21—N8—C20—C23'	161.5 (14)
Ni1—N1—C1—C4	−17.0 (17)	C20—N8—C21—C22	0.7 (6)
C3—N1—C1—C4'	−161 (3)	C20—N8—C21—C25	−179.0 (4)
Ni1—N1—C1—C4'	8(3)	N8—C21—C22—N7	−0.6 (5)
C2—N2—C1—N1	0.4 (6)	C25—C21—C22—N7	179.1 (5)
C2—N2—C1—C4	−174.9 (15)	N8—C21—C22—C26	−179.2 (4)
C2—N2—C1—C4'	161 (3)	C25—C21—C22—C26	0.5 (9)
C1—N2—C2—C3	−0.1 (5)	C20—N7—C22—C21	0.2 (5)
C1—N2—C2—C6	−179.7 (4)	Ni1—N7—C22—C21	174.8 (3)
N2—C2—C3—N1	−0.3 (5)	C20—N7—C22—C26	179.0 (4)
C6—C2—C3—N1	179.2 (5)	Ni1—N7—C22—C26	−6.4 (5)
N2—C2—C3—C13	179.5 (4)	N7—C20—C23—C24	138.9 (17)
C6—C2—C3—C13	−0.9 (9)	N8—C20—C23—C24	−59 (3)
C1—N1—C3—C2	0.6 (5)	C23'—C20—C23—C24	43 (4)
Ni1—N1—C3—C2	−169.4 (3)	N7—C20—C23'—C24'	−134.0 (15)
C1—N1—C3—C13	−179.3 (4)	N8—C20—C23'—C24'	66 (2)
Ni1—N1—C3—C13	10.7 (6)	C23—C20—C23'—C24'	−31 (4)
N1—C1—C4—C5	134 (2)	C15—C16—C26—C22	123.5 (6)
N2—C1—C4—C5	−52 (3)	N5—C16—C26—C22	−55.9 (6)
C4'—C1—C4—C5	48 (17)	C21—C22—C26—C16	−125.9 (5)
N1—C1—C4'—C5'	−164 (5)	N7—C22—C26—C16	55.6 (5)

### Hydrogen-bond geometry (Å, °)

**Table:**

*D*—H···*A*	*D*—H	H···*A*	*D*···*A*	*D*—H···*A*
N2—H2···O4^i^	0.86	2.29	3.12 (2)	162
N2—H2···O5'^i^	0.86	1.88	2.67 (5)	152
N4—H4···O2^ii^	0.86	2.37	3.063 (5)	138
N6—H6···O6^iii^	0.86	2.05	2.83 (2)	152
N6—H6···O4'^iii^	0.86	2.18	2.98 (3)	155
N8—H8···O5^iv^	0.86	2.01	2.81 (3)	155

Symmetry codes: (i) −*x*+1, −*y*, −*z*+1; (ii) −*x*+3/2, *y*+1/2, −*z*+3/2; (iii) −*x*+1, −*y*+1, −*z*+1; (iv) −*x*+1, *y*, −*z*+3/2.
